# The dynamic interrelationships of diabetes distress uncovered by ecological momentary assessment: a systematic review

**DOI:** 10.3389/fendo.2026.1793398

**Published:** 2026-03-26

**Authors:** Shili Yang, Yulong Yang, Mingli Li, Shuang Hu, Xiangrong Liu

**Affiliations:** 1 College of Nursing, Changchun University of Chinese Medicine, Changchun, China; 2 Department of Pharmacy, The Third Affiliated Hospital of Changchun University of Chinese Medicine, Changchun, China

**Keywords:** diabetes distress, dynamic monitoring, ecological momentary assessment, self-management, systematic review

## Abstract

**Objective:**

This systematic review aimed to synthesize evidence on the application of Ecological Momentary Assessment (EMA) in dynamically monitoring diabetes distress, and to examine the dynamic associations between EMA-measured psychosocial or contextual factors and diabetes distress.

**Methods:**

A systematic search of multiple English and Chinese databases was conducted up to January 13, 2026. Studies were eligible if they employed EMA to dynamically monitor diabetes distress and related psychosocial or contextual factors in individuals with diabetes. The methodological quality of included studies was assessed using the Newcastle-Ottawa Scale.

**Results:**

Seventeen studies involving a total of 1,807 participants (predominantly with type 1 diabetes) were included. The average EMA completion rate was 84.1%, with lower rates observed among adolescents compared to adults. Subjective glucose perception, the nature of social interactions, sedentary behavior, and physical symptoms showed momentary associations with diabetes distress. Distress was found to prospectively predict subsequent lapses in self-management behaviors and exacerbate negative emotional states.

**Conclusion:**

EMA is a feasible tool for assessing diabetes distress, effectively capturing its dynamic nature and real-time links with contextual factors. It provides a reference for developing timely and personalized psychological interventions in diabetes care.

**Systematic Review Registration:**

https://www.crd.york.ac.uk/PROSPERO/view/CRD420251156226, identifier CRD420251156226.

## 1 Introduction

The global prevalence of diabetes mellitus (DM) continues to rise alongside an aging population, currently affecting approximately 588.7 million individuals worldwide (1). Diabetes significantly impairs quality of life, long-term health outcomes, and imposes a substantial psychological burden. Among various psychosocial factors, diabetes distress has emerged as a major concern following depression, due to its high prevalence and detrimental effects (2). Diabetes distress refers to the disease-specific emotional response arising from the challenges of diabetes management, the threat of complications, and perceived compromised quality of life. Its core features include frustration, fear, worry, and a sense of overwhelming treatment burden. Consequently, effective identification and intervention for diabetes distress have become integral to comprehensive diabetes care.

Traditional assessment of diabetes distress primarily relies on retrospective self-report questionnaires, such as the Diabetes Distress Scale (DDS) and the Problem Areas in Diabetes (PAID) scale. These tools require individuals to summarize their emotional experiences over a past period, a process susceptible to recall bias, momentary mood fluctuations, and cognitive reconstruction (3, 4). Moreover, diabetes distress is not a stable trait but exhibits dynamic fluctuations. Traditional questionnaires are ill-equipped to capture these dynamic features and real-time associations with contextual factors, thereby limiting the understanding of underlying mechanisms and the development of clinical interventions.

Ecological Momentary Assessment (EMA) is an emerging methodology for real-time, high-frequency data collection. It leverages mobile technology to repeatedly and dynamically capture an individual’s momentary states, behaviors, and environmental contexts within their natural environment (5). EMA effectively overcomes recall bias inherent in retrospective measures and can reveal the micro-processes of emotion-behavior interactions (6). While previous reviews have acknowledged the potential of EMA in diabetes research, they have often focused on broader psychosocial aspects or specific behaviors like physical activity (7). To our knowledge, no systematic review has yet synthesized EMA evidence specifically on diabetes distress as a primary outcome. Furthermore, existing work has not integrated these findings into a cohesive dynamic framework that captures the real-time interplay between distress, its momentary triggers, and its immediate consequences. Therefore, this systematic review aimed to evaluate the feasibility of EMA for dynamically monitoring diabetes distress, synthesize evidence on its dynamic associations with momentary antecedents and proximal consequences, and provide insights for the scientific monitoring, assessment, and intervention of diabetes distress.

## 2 Methods

This study was guided by the Preferred Reporting Items for Systematic Reviews and Meta-Analyses (PRISMA) statement. The review protocol was pre-registered online in PROSPERO (#CRD420251156226).

### 2.1 Inclusion and exclusion criteria

#### 2.1.1 Inclusion criteria

1. Patients with a clinical diagnosis of type 1 or type 2 diabetes.
2. Studies employing EMA as the core data collection method. The EMA protocol must include real-time or near-real-time assessment of diabetes distress, its core dimensions, or related psychological constructs, involving repeated measurements in natural environments. EMA protocols can be categorized by their sampling strategies. Signal-contingent sampling requires participants to respond to a signal (e.g., an alarm or notification) at predetermined or random times. Event-contingent sampling requires participants to initiate a report whenever a predefined event of interest occurs (e.g., a meal or a social interaction). Interval-contingent sampling requires participants to complete assessments at regular, predetermined intervals (e.g., every evening at 8 PM).
3. Studies must clearly describe the specific EMA implementation for measuring diabetes distress and fully report dynamic quantitative outcome measures based on EMA (e.g., momentary levels, day-to-day variability, associations with antecedent/consequent variables).
4. Study designs including cross-sectional studies, prospective or retrospective cohort studies, and case-crossover designs.

#### 2.1.2 Exclusion criteria

1. Review articles, systematic reviews, meta-analyses, conference abstracts, animal studies, theses, case reports, and qualitative studies.
2. Studies for which the full text could not be obtained despite exhaustive efforts (database retrieval, contacting authors, inter-library loan).
3. Non-English or non-Chinese literature where full text or key data could not be accessed via translation services.

### 2.2 Search strategy

A comprehensive search strategy was employed to minimize the risk of omitting relevant literature. We systematically searched the following electronic databases from their inception to January 13, 2026: PubMed, EMBASE, the Cochrane Central Register of Controlled Trials, Web of Science, China National Knowledge Infrastructure (CNKI), Wanfang Data, VIP, and Chinese Biomedical Literature Database (CBM). Preliminary searches indicated that mandating the specific term “diabetes distress” would lead to the omission of relevant studies due to significant heterogeneity in terminology within this research field. Therefore, the final search strategy was constructed around two core concepts: “diabetes” AND “ecological momentary assessment.” We utilized a combination of controlled vocabulary (e.g., MeSH terms) and free-text terms tailored to each database. The search strategy for PubMed is an example (**Table 1**). During the full-text screening phase, we further identified and included studies that, while not explicitly focused on diabetes distress, examined closely related constructs relevant to our review.

**Table 1:** Search strategy for PubMed.

Search	Query
#1	“Diabetes Mellitus”[MeSH Terms]
#2	“Diabetes Mellitus Experimental”[Title/Abstract] OR “Diabetes Mellitus type 1”[Title/Abstract] OR “Wolfram Syndrome”[Title/Abstract] OR “Diabetes Mellitus type 2”[Title/Abstract] OR “Diabetes Mellitus Lipoatrophic”[Title/Abstract] OR “Donohue Syndrome”[Title/Abstract] OR “Latent Autoimmune Diabetes in Adults”[Title/Abstract]
#3	#1 OR #2
#4	“Ecological Momentary Assessment”[MeSH Terms]
#5	“Assessments Ecological Momentary”[Title/Abstract] OR “Ecological Momentary Assessments”[Title/Abstract] OR “Experience Sampling”[Title/Abstract] OR “Experience Samplings”[Title/Abstract] OR “Experience Sampling Method”[Title/Abstract] OR “Ambulatory Assessment”[Title/Abstract] OR “Real-time Data Capture”[Title/Abstract] OR “Diary Study”[Title/Abstract] OR “EMA”[Title/Abstract] OR “ESM”[Title/Abstract]
#6	#4 OR #5
#7	#3 AND #6

### 2.3 Study selection and data extraction

Two trained reviewers independently performed study selection and data extraction based on the pre-defined criteria. Discrepancies were resolved through discussion with a third reviewer. After removing duplicates using NoteExpress software, titles and abstracts were screened, followed by full-text assessment of potentially eligible studies. Extracted data included: country, study design, sample size, diabetes type, mean disease duration, baseline HbA1c, EMA device type, total EMA assessment duration, daily assessment frequency, diabetes distress measurement items, EMA completion rate, momentary antecedent variables, and proximal consequence variables of diabetes distress.

### 2.4 Quality assessment

Two reviewers independently assessed the methodological quality of included observational studies using the Newcastle-Ottawa Scale (NOS) (8). The NOS comprises 8 items across three domains: selection, comparability, and exposure/outcome. Total scores range from 0 to 9, with 0-3, 4-6, and ≥7 indicating low, moderate, and high quality, respectively.

## 3 Results

### 3.1 Literature search results

**Figure 1:**
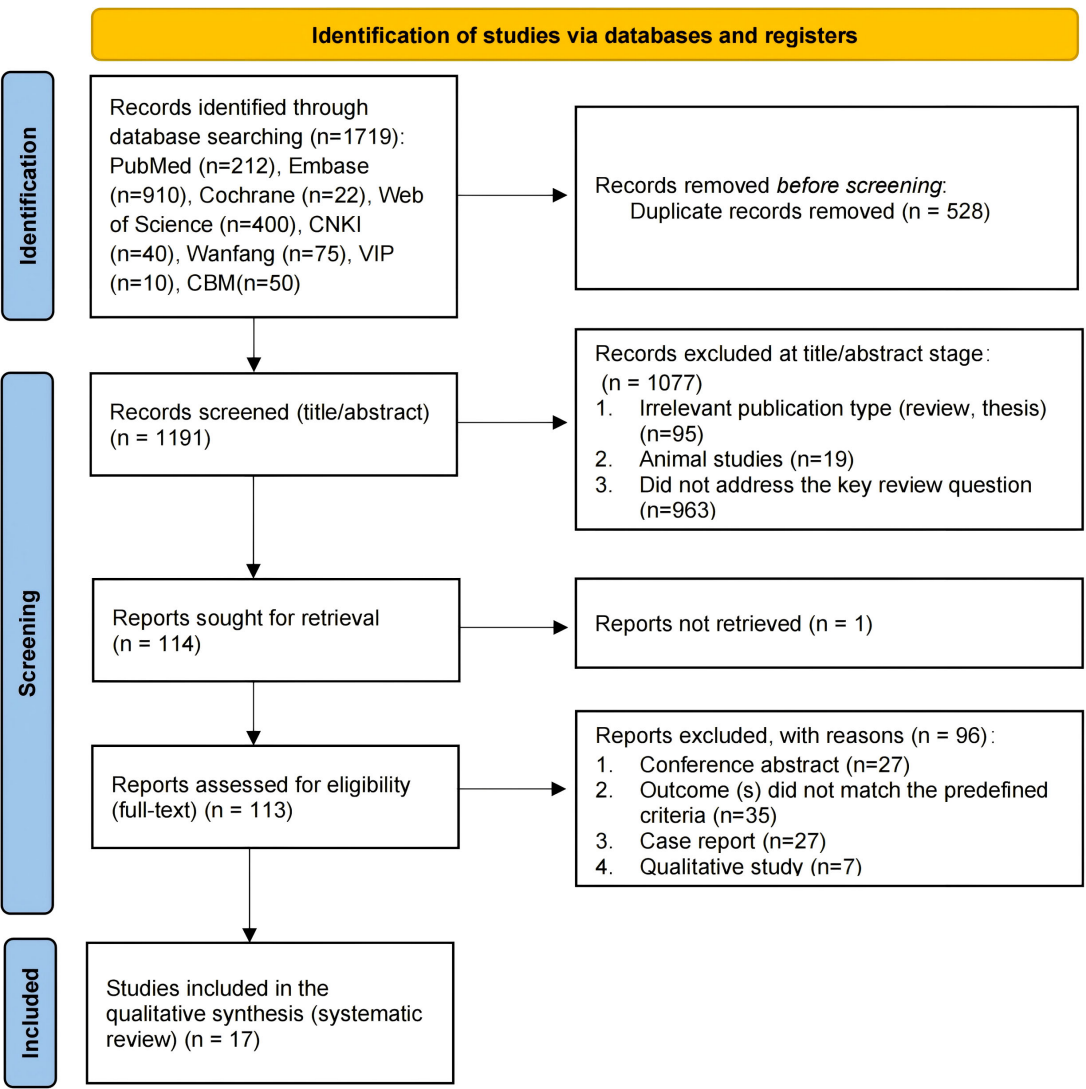
PRISMA flow diagram of the study selection process. The diagram outlines the number of records identified through database searching and other sources, the number of duplicates removed, and the sequential stages of screening (titles/abstracts and full-text) with reasons for exclusion at each stage, culminating in the final number of studies included in the qualitative synthesis.

The initial search yielded 1719 records. After removing 528 duplicates, 1191 unique records were screened by title and abstract. Of these, 1077 were excluded, leaving 114 articles for full-text review. The full text of one article could not be retrieved. After assessing the remaining 113 articles, 17 studies met the pre-defined inclusion and exclusion criteria and were included in this systematic review. The detailed selection process and reasons for exclusion are presented in the PRISMA flow diagram (**Figure 1**).

### 3.2 Characteristics of included studies and EMA implementation

Seventeen observational studies with an average quality score of 7 (SD = 1.01) on the NOS were included, encompassing a total sample size of 2,496 individuals as reported in the original publications. It is worth noting that several included studies originated from the same cohorts. Specifically, Pham et al. (9), Gonzalez, Hoogendoorn et al. (10), and Gonzalez, Schneider et al. (11) were based on the FEEL-T1D cohort (N = 182). Similarly, Ehrmann et al. (12) and Hermanns et al. (13) were based on the DIA-LINK1 cohort (N = 193). Ehrmann et al. (14) included participants from the DIA-LINK1 cohort along with a newly recruited type 2 diabetes cohort (hereafter referred to as DIA-LINK2, N = 186); the type 2 diabetes sample of Hermanns et al. (13) was also based on this same DIA-LINK2 cohort. Despite these cohort overlaps, each study reported distinct outcomes or conducted different analyses, and thus all were retained in this review. After accounting for these overlaps, the 17 publications represent 14 unique observational studies, with a total of 1,807 unique participants. The majority of studies (n = 13, 76.5%) focused primarily on individuals with type 1 diabetes. EMA implementation protocols were heterogeneous (**Table 2**). Four studies (23.5%) employed event-contingent sampling to capture daily fluctuations (15–18), one study used interval-contingent sampling (19), and the remaining studies (70.6%) used signal-contingent sampling (9, 10, 12–14, 20–26). The median assessment period was 14 days (range: 3–70 days), with a median of 4 daily prompts (range: 1–24 prompts). For the measurement of distress, 47.1% of studies utilized a single item or a visual analog scale for rapid assessment to minimize participant burden. The remaining studies employed adapted or shortened versions of standardized multi-dimensional scales to capture richer information. Smartphone applications were the predominant technological platform (88.2%, n=15 studies). Nine studies (52.9%) achieved temporal synchronization between EMA and continuous glucose monitoring (CGM) data, enabling the real-time pairing of psychological states with physiological glucose metrics (10–15, 17, 25, 26).

**Table 2:** Summary of basic characteristics and methodological quality of the included studies.

Author/year	Country	Sample characteristics	Mean disease duration (Years)	Baseline HbA1c (%)	EMA device type	Total EMA duration (Days)	Daily prompts	Assessment dimensions	EMA completion rate (%)	Quality (NOS)
Mulvaney et al., 2019 (16)	USA	T1D, N = 30; 15.40 ± 1.52 yrs; 53.3% F	5.96 ± 4.41	8.00 ± 1.16	Smartphone app	30	3	Perceived stress, negative affect, energy/fatigue, management barriers, social/environmental context	55	6
Shapira et al., 2023 (22)	USA	T1D, N = 32; 16.60 ± 1.40 yrs; 56.3% F	8.80 ± 4.20	8.80 ± 1.40	Smartphone app	14	4	Negative affect, guilt/criticism, illness-related cognitions	72	6
Wooldridge et al., 2022 (20)	USA	T2D, N = 10; 54.00 ± 4.20 yrs; 20.0% F	9.80 ± 6.90	7.20 ± 1.80	Smartphone app	14	6	Overall distress, management barriers	96	7
Horner et al., 2025 (26)	USA	T1D, N = 88; 15.67 ± 0.78 yrs; 45.5% F	NR	8.40 ± 1.42	Smartphone app	8	8	Negative affect	99	7
Gonzalez et al., 2025 (10)	USA	T1D, N = 182; 40.10 ± 14.50 yrs; 53.8% F	20.70 ± 12.80	NR	Smartphone app	14	5-6	Perceived stress	95	9
Søholm et al., 2024 (25)	USA	T1D, N = 274; 44.9 yrs; 54.0% F T2D, N = 320; 61.9 yrs; 36.9% F	T1D: 23.80 ± 15.60 T2D: 20.40 ± 8.90	T1D: 7.30 ± 0.90 T2D: 7.70 ± 1.30	Smartphone app	70	3	Overall distress, glucose burden, physical symptoms, self-management, quality of life, social context	91	9
Poppe et al., 2021 (18)	Belgium	T2D, N = 38; 63.18 ± 7.80 yrs; 34.2% F	10.80 ± 6.90	NR	Smartphone app	10	1	Physical symptoms, negative affect, self-management	90	7.5
Moskovich et al., 2019 (17)	USA	T1D, N = 83; 41.90 yrs (18–68); 88.0% F	23.40 ± 13.40	8.80 ± 2.30	IfByPhone^®^ system	3	12–24	Overall distress, negative affect, self-management	96	7
Zhang et al., 2022 (21)	USA	T1D, N = 45; 13.30 ± 1.70 yrs; 53.3% F	5.50 ± 3.70	9.00 ± 1.90	Smartphone app	30	4	Social context, physical symptoms, negative affect, self-management, stress	50	7.5
Hermanns et al., 2025 (13)	Germany	T1D, N = 192; 38.70 ± 12.70 yrs; 58.3% F T2D, N = 179; 53.30 ± 9.40 yrs; 41.3% F	T1D: 18.90 ± 11.60 T2D: 12.00 ± 7.60	T1D: 8.70 ± 1.90 T2D: 9.00 ± 1.70	Smartphone app	8	3	Overall distress, glucose burden, physical symptoms, self-management, quality of life, social context	71	8
Ehrmann et al., 2022 (12)	Germany	T1D, N = 178; 38.60 yrs (18-70); 59.0% F	19.00 ± 11.70	8.60 ± 1.90	Smartphone app	17	1	Overall distress, glucose burden, physical symptoms, self-management, quality of life, social context	79	7
Merwin et al., 2015 (15)	USA	T1D, N = 83; 41.89 ± 12.43 yrs; 88.0% F	23.43 ± 13.39	8.80 ± 2.32	Smartphone app	3	9	Negative affect, overall distress, self-management	96	6
Gonzalez, Schneider et al., 2025 (11)	USA	T1D, N = 182; 40.1 ± 14.5 yrs; 53.8% F	20.7 ± 12.8	8.6 ± 1.9	Smartphone app	14	5-6	Self-efficacy, diabetes distress, self-management, glycemic regulation	95	7
Helgeson et al., 2024 (24)	USA	T1D, N = 167; 15.83 ± 0.78 yrs (14-17); 49.1% F	NR	8.49 ± 1.61	Smartphone app	8	8	Social context, negative affect, self-management	79	7
Helgeson et al., 2009 (19)	USA	T1D, N = 76; 14.54 yrs (13-16); 50.0% F	6.34 ± 3.22	8.65 ± 1.78	PDA	4	6-8	Social context, negative affect, glucose burden, quality of life	88	7
Pham et al., 2023 (9)	USA	T1D, N = 122; 41.12 ± 14.83 yrs; 55.7% F	NR	NR	Smartphone app	14	5-6	Overall distress, negative/positive affect, physical symptoms, social context	93	7
Ehrmann et al., 2024 (14)	Germany	T1D, N = 193; 39.10 ± 12.70 yrs; T2D, N = 186; 52.80 ± 9.70 yrs; 49.6% F	T1D: 18.90 ± 11.90 T2D: 11.90 ± 7.60	T1D: 8.60 ± 1.90 T2D: 9.00 ± 1.70	Smartphone app	17	1	Overall distress, glucose burden	NR	7

T1D, Type 1 Diabetes; T2D, Type 2 Diabetes; NR, Not Reported; F, Female.

### 3.3 Feasibility of EMA and participant burden

The average EMA completion rate across included studies was 84.1%, indicating good feasibility for dynamically monitoring diabetes distress in naturalistic settings. However, completion rates varied considerably between studies, closely related to EMA protocol intensity, measurement tools, and participant characteristics. Studies with protocols involving more than 5 daily prompts or a total duration exceeding 30 days more frequently reported declining completion rates or increased participant burden (15, 17, 21, 25). The complexity of measurement tools was also associated with completion; studies using single-item or VAS measures reported a higher average completion rate (~90.2%) (10, 20) compared to those using multi-item scales (~78.5%) (12, 14). Furthermore, adaptability to EMA varied across populations. Adolescents with diabetes showed lower average completion rates (78-80%) than adult patients (85-96%). This discrepancy between adolescents and adults was consistent across studies that included both age groups, and appears to be a more significant source of variance in completion rates than protocol intensity alone, although longer, more burdensome protocols exacerbated the issue in all populations (27).

### 3.4 Dynamic features of diabetes distress revealed by EMA

#### 3.4.1 Momentary antecedents

##### 3.4.1.1 Subjective cognitive mediation of the glucose-distress link

While one study found that CGM-based glucose variability was not significantly associated with diabetes distress (12), another showed that perceived glucose variability was the strongest predictor (14). This apparent conflict highlights the crucial mediating role of cognitive appraisal, a finding consistently echoed across multiple studies (10, 12, 14). Specifically, a higher percentage of time spent above 180 mg/dL was associated with greater distress (*β* = 0.41) in one study (12), but the impact of such objective metrics appears to be filtered through subjective perception: perceived glucose variability (*t* = 14.360; *p* < 0.0001) and perceived hyperglycemia (*t* = 13.637; *p* < 0.0001) were the strongest predictors of daily distress in another (14).

This notion is further supported by findings from the Hypo-RESOLVE study, which showed that only subjectively reported hypoglycemia, and not all objectively recorded events, had a significant negative impact on daily functioning and mood. Notably, even “prevented” hypoglycemic events—where an individual took corrective action to avoid a low glucose value (<70 mg/dL) but still experienced the subjective burden and fear of an impending hypo—were associated with these negative effects (25).

##### 3.4.1.2 Short-term emotional modulation by social interactions

EMA-based lagged analyses revealed that when the supportiveness rating of a peer interaction was above an individual’s own average, their subsequent positive affect significantly increased (*β* = 0.29, *p* < 0.001) and negative affect decreased (*β* = -0.06, *p* < 0.001). Conversely, interactions rated higher in conflict predicted decreased subsequent positive affect (*β* = -0.17, *p* < 0.001) and increased negative affect (*β* = 0.23, *p* < 0.001) (24). These findings are consistent with another EMA study showing that social context is closely linked to momentary emotional states in adolescents with type 1 diabetes (19).

##### 3.4.1.3 Concurrent fluctuations of sedentary behavior, physical sensations, and distress

EMA monitoring identified covariance between sedentary behavior and diabetes distress. Reports of sedentary behavior were associated with significantly higher concurrent diabetes distress (*r* = 0.02, *p* < 0.001) (9). Additionally, Hermanns et al. (13). found that higher baseline diabetes distress (measured by the retrospective PAID questionnaire) was associated with elevated reporting of nearly all somatic symptoms during the EMA period, suggesting that trait-level distress may heighten sensitivity to physical sensations.

#### 3.4.2 Proximal consequences

##### 3.4.2.1 Immediate impact on self-management behaviors

Time-series analyses based on EMA confirmed that each unit increase in negative affect was associated with a 17% decrease in the odds of checking blood glucose (*OR* = 0.83), an effect more pronounced in adolescents with poorer glycemic control (HbA1c >8%; *OR* = 0.75) (22). Other studies reported similar patterns, where distress or high-stress states predicted missed glucose checks or insulin injections in the subsequent hours (16, 21). Distress has also been linked to an increased risk of subsequent binge eating (17).

##### 3.4.2.2 Depletion of emotional state and psychological resources

Diabetes distress precipitates a cluster of negative emotions and is linked to long-term psychological risk. EMA’s high-frequency assessment reveals that diabetes distress itself constitutes a multifaceted negative emotional state encompassing frustration, worry, and fear (15). More importantly, this subjective experience has predictive utility over time. Using n-of-1 analyses, Ehrmann et al. (14) examined the strength of the associations between daily diabetes distress and both perceived and objective glucose metrics for each individual. They found that individuals with a stronger association between perceived glucose variability and daily distress—i.e., those whose distress fluctuated more closely with their subjective perception of glucose variability—had more severe depressive symptoms, diabetes distress, and fear of hypoglycemia three months later (*p* < 0.001). Similarly, individuals with a stronger association between perceived hyperglycemia and daily distress also reported more depressive symptoms at follow-up (*p* = 0.02). In contrast, individuals for whom objective CGM metrics (e.g., time in hypoglycemia) were more strongly associated with daily distress showed better psychosocial outcomes at follow-up, including fewer depressive symptoms, lower diabetes distress, and less fear of hypoglycemia (*p* < 0.01), but also higher HbA1c (*p* < 0.05). Notably, perceived glucose burden alone did not directly predict these outcomes at follow-up. This pattern suggests that it is not merely the level of perceived glucose burden itself, but rather the extent to which an individual’s distress is coupled with their perceptions of glucose fluctuations, that may deplete psychological resources and contribute to long-term emotional deterioration. The differential predictive patterns of perceived versus objective glucose-distress associations highlight the importance of understanding individual-level drivers of diabetes distress.

##### 3.4.2.3 Indirect and lagged effects on glycemia

The effect of diabetes distress on glycemia appears to follow an indirect pathway mediated primarily by self-management behavior, with a noticeable temporal lag. While several studies found no stable contemporaneous association between negative affect and glucose levels (12, 26), distress was significantly correlated with elevated long-term glucose variability (GV) (22). This pattern is supported by recent evidence from studies employing intensive longitudinal designs to examine within-person dynamics. Gonzalez, Hoogendoorn et al. (10) examined bidirectional relationships between glycemia and diabetes distress over 3-hour intervals using EMA and CGM in a diverse sample of adults with type 1 diabetes (N = 182). Their multilevel cross-lagged panel models revealed that higher mean glucose, lower time in range (70–180 mg/dL), greater time in high (181–250 mg/dL) and very high (>250 mg/dL) glucose ranges, and higher glucose variability over a 3-hour period each significantly predicted greater diabetes distress at the end of that interval (all *p* < 0.05). Higher mean glucose over 3 hours also predicted greater diabetes distress 3 hours later (*p* < 0.05). In contrast, diabetes distress did not predict subsequent CGM metrics, with the exception of a small but significant association with less time in hypoglycemia (<70 mg/dL) over the next 3 hours (*p* < 0.05). Exploratory analyses indicated that the associations between glycemia and distress were stronger among individuals who used personal CGM devices as part of their clinical care, suggesting that real-time glucose feedback may heighten emotional responses to glycemic fluctuations. Taken together, these findings suggest that glucose dysregulation tends to precede short-term increases in diabetes distress, rather than the reverse, although distress may trigger behavioral responses that reduce hypoglycemia risk. This pattern aligns with broader evidence that distress likely does not alter glucose directly but may impair self-management behaviors, leading to worsened glycemic control over longer timeframes (11, 28). The moderating effect of CGM use further highlights the role of real-time glucose feedback in shaping emotional responses to glycemia.

In summary, EMA evidence synthesized in this review characterizes diabetes distress dynamically as: (I) a phenomenon exhibiting significant, non-random fluctuations rather than stable traits; (II) fluctuations tightly linked to immediate contextual factors, including subjective appraisal of glucose, social interaction quality, current behavior, and physical sensations; and (III) a state that proximally triggers impairments in self-management and broad negative affect, ultimately influencing glycemic stability via behavior. These findings suggest that diabetes distress may be conceptualized as a dynamic process involving real-time interaction and feedback among physiological, cognitive, behavioral, and social dimensions.

## 4 Discussion

### 4.1 Main findings

This systematic review synthesizes dynamic evidence from EMA studies on diabetes distress. EMA was generally feasible for dynamic monitoring, though completion rates tended to decline when daily prompts exceeded five or the total period surpassed 30 days. EMA-monitored diabetes distress showed significant dynamic associations with subjective glucose perception, social interactions, and behavioral states, and dynamically influenced subsequent self-management and long-term emotional trends. By integrating EMA evidence, this review clarifies the dynamic evolution of diabetes distress and supports the clinical utility and practical application value of EMA as a dynamic monitoring tool.

### 4.2 The dynamic process of diabetes distress

**Figure 2:**
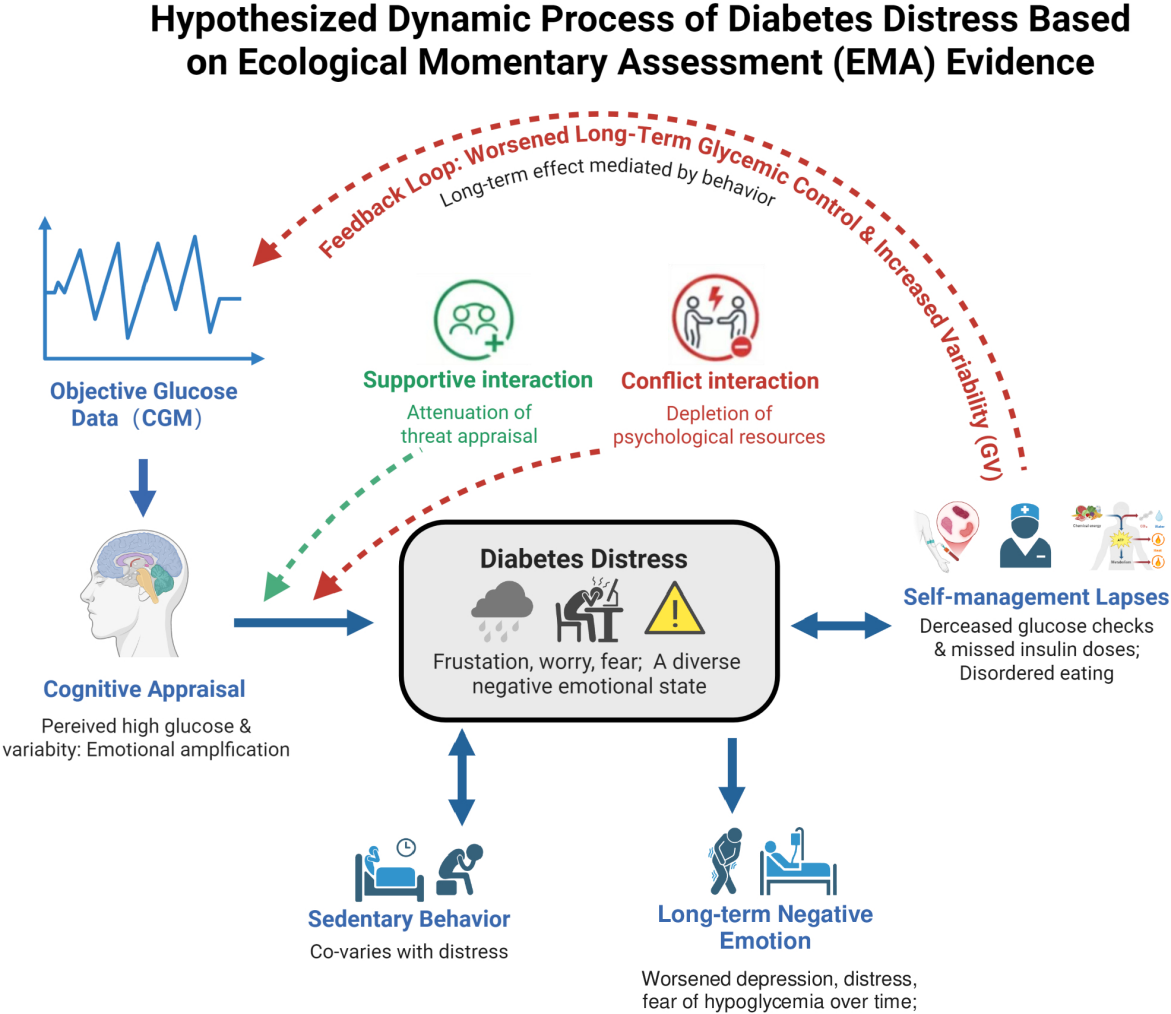
Hypothesized dynamic process of diabetes distress based on EMA, ecological momentary assessment evidence. Bidirectional arrows (↔) represent observed associations and feedback loops. Unidirectional arrows (→) indicate temporal sequences from lagged analyses. The dashed arrow from self-management lapses to objective glucose data represents a hypothesized long-term feedback loop requiring further validation. Dashed moderator arrows indicate the moderating role of social context. This is a heuristic framework, not a causal model.

The integrated EMA evidence supports conceptualizing diabetes distress as a dynamic process involving continuous interaction and feedback among physiological, cognitive, behavioral, emotional, and socio-environmental factors (**Figure 2**). This conceptualization should be viewed as an integrative framework that synthesizes the current evidence, providing a roadmap for future research to empirically examine these proposed pathways, rather than as a definitive causal structure.

The impact of dysglycemia on distress depends on the individual’s subjective cognitive appraisal. This appraisal is closely linked to the immediate experience of distress. This may explain the weak correlation often found between objective glucose and distress (10), as the impact of objective metrics appears to be filtered through subjective perception. This notion is further supported by findings from the Hypo-RESOLVE study, which showed that only subjectively reported hypoglycemia—and even “prevented” hypoglycemic events—had negative effects on daily functioning and mood, whereas objectively recorded events alone did not (25). This state of distress may, in turn, be associated with difficulties in judgment and executive function, which could contribute to lapses or avoidance in key self-management behaviors. Suboptimal self-management may ultimately be related to worsened glycemic control, and these deteriorating physiological metrics may then serve as new stressors, re-entering the cognitive appraisal loop and potentially contributing to heightened distress. This suggests that distress likely does not alter glucose directly and instantaneously but may impair self-management behaviors, leading to worsened glycemic control—an effect that may require hours to days to manifest fully (11, 28). These interrelationships point to a potential reinforcing cycle: “physiological signal → subjective threat appraisal → distress → suboptimal self-management → physiological deterioration.”

Social context plays a key moderating role in this dynamic process. Supportive, understanding social interactions can weaken threat appraisals, enhance positive affect and coping confidence, and thus mitigate the intensity of the vicious cycle. Conversely, conflict-laden, stressful interactions directly exacerbate distress, accelerating the depletion of psychological resources and deepening entrapment in the cycle. This indicates that the social environment, as a proximal contextual factor, can swiftly influence emotional experience, thereby buffering or exacerbating diabetes-related psychological stress (29). Additionally, EMA data show that diabetes distress covaries with daily behavioral patterns. Sedentary behavior has been associated with higher concurrent distress (9), suggesting that behavioral states may influence emotional experience. Furthermore, Hermanns et al. (13) found that higher baseline diabetes distress was associated with elevated reporting of somatic symptoms during the EMA period, indicating that trait-level distress may heighten sensitivity to physical sensations, even if real-time bidirectional associations between symptoms and distress were not examined in that study.

This conceptualization of diabetes distress as a dynamic process aligns with and extends established theoretical frameworks such as the Transactional Model of Stress and Coping (30, 31). In this model, a potential stressor (e.g., a high glucose reading) is subject to primary appraisal (e.g., ‘this is a threat’) and secondary appraisal (e.g., ‘I don’t know how to fix this’), which then elicits an emotional response (distress). Our findings add granularity by specifying the real-time behavioral consequences of this distress and highlighting how these consequences (e.g., missed insulin dose) can become new stressors, thus creating the feedback loop described above. The integration of social context as a crucial moderator is consistent with socio-ecological models of health behavior.

The integration of EMA with CGM provides powerful insights into the bidirectional, time-lagged relationships between affect and glycemia. For instance, Gonzalez et al. (10) used this approach to disentangle the directionality of this association in a diverse sample of adults with type 1 diabetes. Their findings demonstrate that glucose dysregulation—particularly hyperglycemia, reduced time in range, and increased glycemic variability—precedes subsequent increases in diabetes distress over short (3-hour) timeframes, rather than the reverse. While distress was not found to predict subsequent hyperglycemia, it was associated with less time in hypoglycemia in the following hours, possibly reflecting behavioral responses to distress (e.g., increased monitoring or carbohydrate intake). Furthermore, exploratory analyses suggested that the link between glycemia and distress may be amplified in individuals using personal CGM devices, indicating that real-time glucose feedback may heighten awareness and emotional reactivity to glucose fluctuations—a finding that underscores the role of cognitive appraisal in this dynamic process. These findings reinforce the conceptualization that glycemia and distress are not directly linked in a simple, contemporaneous manner, but rather through a complex, temporally dynamic pathway involving behavior and perception—a perspective that aligns with the dynamic process outlined above.

### 4.3 Advantages and challenges of EMA in monitoring diabetes distress

The synthesized evidence indicates that EMA, with its real-time, ecological, and high-density sampling characteristics, not only validates context-mood associations that are elusive to traditional questionnaires but also provides critical theoretical and practical insights into the dynamic evolution of diabetes distress. However, significant heterogeneity in EMA assessment protocols and measurement methods across studies poses challenges for evidence integration. Furthermore, discussions regarding the causes of participant data missingness are generally limited. Such missingness may be non-random and related to the state of distress itself, potentially biasing the conclusions. Future research should, therefore, aim to establish a consensus on core outcome measures and reporting standards to facilitate cross-study synthesis. Study designs must also prioritize participant experience and adherence, striving for an optimal balance between data quality and research burden.

From an ethical standpoint, the intensive and personal nature of EMA data collection necessitates careful consideration. All included studies reported obtaining ethical approval from their respective institutional review boards and informed consent from all participants (and guardians for minors). The high average completion rate (84.1%) suggests that, overall, the perceived burden was acceptable. However, the potential for participant burden, privacy concerns regarding sensitive momentary data, and the risk of exacerbating distress by repeatedly querying negative emotional states are critical ethical considerations for future EMA research. Study designs must proactively mitigate these risks through data encryption, minimizing question length and frequency, and providing resources for participants who may experience distress.

### 4.4 Clinical and practical implications

At the intervention level, the focus could shift from merely reducing distress scores to identifying and interrupting individualized vicious cycles. While these applications are promising, they represent future research directions. The next crucial step will be to design and test digitally-enabled, just-in-time adaptive interventions (JITAIs) based on these dynamic monitoring data, to achieve a complete closed loop from dynamic assessment to personalized intervention. Specifically, future research could explore developing just-in-time cognitive intervention tools embedded in daily life to provide accurate cognitive frameworks the moment patients interpret glucose data. Intelligent response systems could be built to automatically deliver contextualized support resources when conflictual social situations are detected, buffering the immediate emotional impact of social stress. Situational sensing technology could be leveraged to send executive prompts during identified individual high-risk behavioral time windows, assisting patients in overcoming the action initiation difficulties caused by distress. Such precision intervention strategies targeting the dynamic process offer a novel pathway for achieving efficient, personalized diabetes psychological support, pending empirical validation.

### 4.5 Limitations and future directions

This systematic review has several limitations. First, the included studies were predominantly observational. Although EMA data suggest temporal sequences and potential pathways, definitive causal inferences require further validation through experimental or micro-randomized trial designs. Second, the synthesized evidence exhibits a significant population bias, as samples primarily consisted of individuals with type 1 diabetes from Western (e.g., European and North American) populations. The total sample of 1807% participants, while reasonable for a systematic review, is predominantly composed of these groups. This substantially limits the generalizability of our proposed dynamic process to other populations, such as individuals with type 2 diabetes, those from non-Western cultural backgrounds, older adults with different comorbidities, and underrepresented minority groups.

These limitations point to several directions for future research. First, studies should prioritize the inclusion of more diverse and representative patient populations to validate and extend the use of EMA in diabetes distress research, and to adapt the proposed dynamic process accordingly. Second, concurrent efforts should bridge methodological innovation and clinical translation. This includes developing and validating a core, streamlined set of EMA assessment tools suitable for both research and real-world clinical settings. Third, building on dynamic monitoring data, future work should prioritize the design and testing of digitally-enabled, just-in-time adaptive interventions (JITAIs) that can be embedded within clinical workflows. Such an approach would help achieve a complete closed loop from dynamic assessment to personalized intervention, advancing precision mental health in diabetes care.

## Data Availability

The original contributions presented in the study are included in the article/supplementary material. Further inquiries can be directed to the corresponding author.

## References

[1] Duncan BB Magliano DJ Boyko EJ . IDF diabetes atlas 11th edition 2025: global prevalence and projections for 2050. Nephrology Dialysis Transplant. (2025) 41:7–9. doi: 10.1093/ndt/gfaf177. PMID: 40874767

[2] Tang F Guo X Zhang L Yuan L Gan T Wang M . The prevalence of diabetes distress in Chinese patients with type 2 diabetes: a systematic review and meta-analysis. Diabetes Res Clin Pr. (2023) 206:110996. doi: 10.1016/j.diabres.2023.110996. PMID: 37956943

[3] Ahne A Khetan V Tannier X Rizvi MIH Czernichow T Orchard F . Extraction of explicit and implicit cause-effect relationships in patient-reported diabetes-related tweets from 2017 to 2021: deep learning approach. JMIR Med Inf. (2022) 10:e37201. doi: 10.2196/37201. PMID: 35852829 PMC9346561

[4] Nygaard M Willaing I Joensen LE Lindgreen P Stenov V Hessler D . A short-form measure of diabetes distress among adults with type 1 diabetes for use in clinical practice: development and validation of the T1-DDS-7. Diabetes Care. (2023) 46:1619–25. doi: 10.2337/dc23-0460. PMID: 37343387

[5] Moskowitz DS Young SN . Ecological momentary assessment: what it is and why it is a method of the future in clinical psychopharmacology. J Psychiatr Neurosci. (2006) 31:13–20. doi: 10.1139/jpn.0602. PMID: 16496031 PMC1325062

[6] Shiffman S Stone AA Hufford MR . Ecological momentary assessment. Annu Rev Clin Psycho. (2008) 4:1–32. doi: 10.1093/oxfordhb/9780199381708.013.1. PMID: 18509902

[7] Nam S Griggs S Ash GI Dunton GF Huang S Batten J . Ecological momentary assessment for health behaviors and contextual factors in persons with diabetes: a systematic review. Diabetes Res Clin Pr. (2021) 174:108745. doi: 10.1016/j.diabres.2021.108745. PMID: 33713720

[8] Wells GA Shea B O Connell D Peterson J Welch V Losos M . The Newcastle-Ottawa Scale (NOS) for assessing the quality of nonrandomized studies in meta-analyses. (2000). Available online at: http://www.ohri.ca/programs/clinical_epidemiology/oxford.asp

[9] Pham LT Hernandez R Spruijt-Metz D Gonzalez JS Pyatak EA . Movement matters: short-term impacts of physical activity on mood and well-being. J Behav Med. (2023) 46:781–90. doi: 10.1007/s10865-023-00407-9. PMID: 36939975 PMC10026784

[10] Gonzalez JS Hoogendoorn CJ Hernandez R Schneider S Mustafiz F Siddhanta M . Diabetes-related distress and glycemic dysregulation in everyday life with type 1 diabetes: which comes first? Diabetes Care. (2025) 48:1453–60. doi: 10.2337/dc25-0559. PMID: 40554543 PMC12279033

[11] Gonzalez JS Schneider S Hoogendoorn C Nandoo L Pyatak E . Self-efficacy, diabetes distress, self-management, and glycemic regulation: within-person pathways in type 1 diabetes. Ann Behav Med. (2025) 59:kaaf088. doi: 10.1093/abm/kaaf088. PMID: 41236802 PMC12616848

[12] Ehrmann D Schmitt A Priesterroth L Kulzer B Haak T Hermanns N . Time with diabetes distress and glycemia-specific distress: new patient-reported outcome measures for the psychosocial burden of diabetes using ecological momentary assessment in an observational study. Diabetes Care. (2022) 45:1522–31. doi: 10.2337/figshare.19669101. PMID: 35613338

[13] Hermanns N Ehrmann D Kulzer B Klinker L Haak T Schmitt A . Somatic and mental symptoms associated with dysglycemia, diabetes-related complications and mental conditions in people with diabetes: assessments in daily life using continuous glucose monitoring and ecological momentary assessment. Diabetes Obes Metab. (2025) 27:61–70. doi: 10.1111/dom.15983. PMID: 39375863 PMC11618240

[14] Ehrmann D Hermanns N Schmitt A Klinker L Haak T Kulzer B . Perceived glucose levels matter more than CGM-based data in predicting diabetes distress in type 1 or type 2 diabetes: a precision mental health approach using n-of-1 analyses. Diabetologia. (2024) 67:2433–45. doi: 10.1007/s00125-024-06239-9. PMID: 39078490 PMC11519212

[15] Merwin RM Dmitrieva NO Honeycutt LK Moskovich AA Lane JD Zucker NL . Momentary predictors of insulin restriction among adults with type 1 diabetes and eating disorder symptomatology. Diabetes Care. (2015) 38:2025–32. doi: 10.2337/dc15-0753. PMID: 26384389 PMC4876774

[16] Mulvaney SA Vaala SE Carroll RB Williams LK Lybarger CK Schmidt DC . A mobile app identifies momentary psychosocial and contextual factors related to mealtime self-management in adolescents with type 1 diabetes. J Am Med Inform Assn. (2019) 26:1627–31. doi: 10.1093/jamia/ocz147. PMID: 31529065 PMC6857499

[17] Moskovich AA Dmitrieva NO Babyak MA Smith PJ Honeycutt LK Mooney J . Real-time predictors and consequences of binge eating among adults with type 1 diabetes. J Eat Disord. (2019) 7:7. doi: 10.1186/s40337-019-0237-3. PMID: 30923613 PMC6421642

[18] Poppe L De Paepe AL Van Ryckeghem DML Van Dyck D Maes I Crombez G . The impact of mental and somatic stressors on physical activity and sedentary behavior in adults with type 2 diabetes mellitus: a diary study. PeerJ. (2021) 9:e11579. doi: 10.7717/peerj.11579. PMID: 34178463 PMC8216170

[19] Helgeson VS Lopez LC Kamarck T . Peer relationships and diabetes: retrospective and ecological momentary assessment approaches. Health Psychol. (2009) 28:273–82. doi: 10.1037/a0013784. PMID: 19450032 PMC2792470

[20] Wooldridge JS Soriano EC Harris DE Afari N . Feasibility and acceptability of ecological momentary assessment of psychosocial factors and self-management behaviors among veterans with type 2 diabetes. Diabetes Spectr. (2022) 35:76–85. doi: 10.2337/ds21-0020. PMID: 35308149 PMC8914587

[21] Zhang P Fonnesbeck C Schmidt DC White J Kleinberg S Mulvaney SA . Using momentary assessment and machine learning to identify barriers to self-management in type 1 diabetes: observational study. JMIR Mhealth Uhealth. (2022) 10:e21959. doi: 10.2196/21959. PMID: 35238791 PMC8931646

[22] Shapira A Volkening LK Borus JS Laffel LM . Ecological momentary assessment of positive and negative affect and associations with blood glucose in teens with type 1 diabetes. J Diabetes Sci Technol. (2023) 17:195–200. doi: 10.1177/19322968211035451. PMID: 34330178 PMC9846400

[23] de Wit M van Raalte DH van den Berg K Racca C Muijs LT Lutgers HL . Glucose variability and mood in people with type 1 diabetes using ecological momentary assessment. J Psychosom Res. (2023) 173:111477. doi: 10.1016/j.jpsychores.2023.111477. PMID: 37643560

[24] Helgeson VS Horner FS Reis HT Niezink NMD Libman I . Peer interactions and health among youth with diabetes: an ecological momentary assessment. Health Psychol. (2024) 43:684–93. doi: 10.1037/hea0001393. PMID: 38635187 PMC11368131

[25] Søholm U Broadley M Zaremba N Divilly P Baumann PM Mahmoudi Z . The impact of hypoglycemia on daily functioning among adults with diabetes: a prospective observational study using the Hypo-METRICS app. Diabetologia. (2024) 67:2160–74. doi: 10.1007/s00125-024-06233-1, PMID: 39080044 PMC11447150

[26] Horner FS Helgeson VS . Glucose in the dynamic psychosocial environment: findings from adolescents with type 1 diabetes. Biopsychosoc Sci Med. (2025) 87:190–6. doi: 10.1097/psy.0000000000001373. PMID: 40632075

[27] Bhave SY Sovani AV Shah SR . Role of psychologist in adolescent medicine: an international perspective. Pediatr Clin N Am. (2022) 69:847–64. doi: 10.1016/j.pcl.2022.05.001. PMID: 36207097 PMC9531961

[28] AlShezawi IA Rawwad THA Aldirawi AA Alwawi AA Fazari HSA Shah AH . Exploring the impact of self-efficacy on glycemic control in Omani type 2 diabetes patients. Front Endocrinol. (2025) 16:1597274. doi: 10.3389/fendo.2025.1597274. PMID: 40786180 PMC12331482

[29] Gan Y Tian F Fan X Wang H Zhou J Yang N . A study of the relationship between social support, depression, alexithymia and glycemic control in patients with type 2 diabetes mellitus: a structural equation modeling approach. Front Endocrinol. (2024) 15:1390564. doi: 10.3389/fendo.2024.1390564. PMID: 39229377 PMC11368761

[30] Folkman S . Personal control and stress and coping processes: a theoretical analysis. J Pers Soc Psychol. (1984) 46:839–52. doi: 10.1037/0022-3514.46.4.839. PMID: 6737195

[31] Steffen PR Anderson T . Primary appraisal is affective not cognitive: exploring a revised transactional model of stress and coping. Appl Psychophys Biof. (2025) 50:197–211. doi: 10.1007/s10484-025-09699-w. PMID: 40056329

